# Supplementation of Omega 3 during Pregnancy and the Risk of Preterm Birth: A Systematic Review and Meta-Analysis

**DOI:** 10.3390/nu13051704

**Published:** 2021-05-18

**Authors:** Ramón Serra, Reyna Peñailillo, Lara J. Monteiro, Max Monckeberg, Macarena Peña, Lía Moyano, Camila Brunner, Georgina Vega, Mahesh Choolani, Sebastián E. Illanes

**Affiliations:** 1 Hospital FF.AA Cirujano Guzmán, Punta Arenas 6212631, Chile; rserratagle@yahoo.com; 2 Department of Obstetrics, Gynecology and Laboratory of Reproduction, Universidad de los Andes, Santiago 7620001, Chile; rpenailillo@uandes.cl (R.P.); lmonteiro@uandes.cl (L.J.M.); mmonckebergz@uandes.cl (M.M.); mspena@miuandes.cl (M.P.); lgmoyano@miuandes.cl (L.M.); cpbrunner@miuandes.cl (C.B.); gsvega@miuandes.cl (G.V.); 3 Department of Obstetrics & Gynaecology, Yong Loo Lin School of Medicine, National University of Singapore, Singapore 119228, Singapore; obgmac@nus.edu.sg; 4 Department of Obstetrics & Gynaecology, National University Hospital, Singapore 119228, Singapore

**Keywords:** omega 3 supplementation, preterm birth, meta-analysis

## Abstract

Preterm birth (PTB) is a major cause of neonatal death and long-term consequences for the newborn. This review aims to update the evidence about the potential benefit of pharmacological supplementation with omega 3 fatty acids during pregnancy on the incidence of PTB. The Medline, Embase, Cochrane Library and Central databases were searched until 28 June 2020 for RCTs in which omega 3 supplementation was used versus placebo to reduce PTB risk. Data from 37 trials were analyzed. We found an 11% reduction in PTB risk (RR(risk ratios), 0.89; 95% CI (confidence intervals), 0.82 to 0.97) in trials using omega 3 supplements versus placebo. Regarding early PTB (ePTB), there was a 27% reduction in the risk of ePTB (RR, 0.73; 95% CI, 0.58 to 0.92). However, after sensitivity analyses, there were no significant differences in PTB and ePTB risk (PTB RR, 0.92; 95% CI, 0.83 to 1.01, ePTB RR, 0.82; 95% CI, 0.61 to 1.09). We conclude that omega 3 supplementation during pregnancy does not reduce the risk of PTB and ePTB. More studies are required to determine the effect of omega 3 supplementations during pregnancy and the risk of detrimental fetal outcomes.

## 1. Introduction

Preterm birth (PTB), defined as birth <37 weeks of gestational age (GA), has been estimated to affect one in nine infants worldwide and represents the most significant cause of neonatal death [1]. Likewise, early PTB (ePTB, birth <34 weeks of GA) also represents a high risk of heart failure, infections and neonatal mortality [2]. Additionally, women who give birth before 37 weeks of GA have an increased risk of preterm deliveries in their second pregnancies [3]. Many strategies have been used to reduce the risk of PTB, particularly, the use of progesterone and cervical cerclage, which have limitations such as cost, availability and safety [4]. One strategy focused on the prevention of PTB is the administration of long-chain polyunsaturated fatty acids (LCPUFA) during pregnancy, which is accessible and cheap and has minimal side effects.

Omega 3 LCPUFA, including eicosapentaenoic acid (EPA) and docosahexaenoic acid (DHA), are essential in metabolic and physiological processes during embryonic and fetal development [5]. Although higher intakes of DHA and EPA’s have been recommended worldwide, Dietary Reference Intakes (DRIs) have not been established. The Institute of Medicine (IOM) recommends that the intake of EPA and DHA contributes to 10 percent of the total omega 3 fatty acid intake (160 mg per day approximately) [6]. In the case of the Food and Agriculture Organization (FAO), the recommendation is an intake of 200 mg/day of DHA during pregnancy [7].

Lower plasma levels of omega 3 LCPUFAs during pregnancy have been associated with detrimental perinatal outcomes, such as preterm delivery [8] and preeclampsia [9]. However, until now, there is no consensus on whether omega 3 supplementation during pregnancy reduces the risk of prenatal complications. A significant number of studies have been carried out to address this topic, including randomized controlled trials (RCTs) and meta-analyses. Some of them have shown a significant association between the consumption of fish or DHA supplements in pregnancy and decreased PTB incidence [10,11,12], but others have failed to demonstrate this association [13]. In 2010, Makrides et al. conducted an RCT to evaluate the impact of DHA supplementation during pregnancy on maternal depression and concluded that the consumption of DHA was associated with a decrease in the incidence of ePTB compared to the control group [14]. However, this outcome was secondary, and the analyses were not adjusted for multiplicity, limiting the confidence in the results. Olsen et al. showed in an RCT that fish oil does not prevent PTB in Chinese women; however, it is important to consider that supplementation started at midgestation [15]. Among the mechanisms suggested to explain the effect of omega 3 on PTB are the production of eicosanoids involved in the parturition process [16] and their role in the inflammatory pathway, including an increase in resolving R3 production [17,18]. Besides its tocolytic properties, omega 3 may also have an effect on the electrical activity of the myometrium during the pre-labor period, demonstrating an antiarrhythmic effect that could explain the role of omega 3 in the prevention of PTB [19].

The primary outcome of this systematic review and meta-analysis is to update the evidence in order to evaluate the potential benefit of pharmacological supplementation with omega 3 fatty acids during pregnancy in the incidence of PTB. We also review the current evidence in order to analyze the potential impact of this supplementation on other perinatal outcomes.

## 2. Materials and Methods

The systematic review and meta-analysis were conducted according to the Cochrane Handbook for Systematic Reviews of Interventions [20].

### 2.1. Eligibility Criteria

The eligibility criteria comprised of RCTs including patients of any age with single or multiple pregnancies, with or without known pathology, and comparing the use of omega 3 fatty acid supplements versus placebo or no intervention, or the use of omega 3 acid supplements in combination with another agent versus placebo or no intervention.

### 2.2. Outcomes

The primary outcome was preterm delivery <37 weeks of gestation; the secondary outcomes included early preterm delivery <34 weeks of gestation, preeclampsia, intrauterine growth restriction (IUGR), fetal death in utero and neonatal death.

### 2.3. Literature Search and Selection of Studies

The search was conducted until 28 June 2020. We searched the following electronic databases: the Cochrane Central Register of Controlled Trials (CENTRAL), the Cochrane Library, MEDLINE (PubMed) and Embase. Moreover, we manually searched for relevant journals, conference proceedings and reference lists. We did not restrict the search by language, date, or publication status in order to reduce publication and retrieval bias.

### 2.4. Data Collection

Two reviewers (R.S. and S.E.I.) independently assessed the results of the electronic search. First, we identified duplicate articles in electronic databases, as well as duplicate articles in different journals. We then identified and selected potentially eligible studies based on title and then abstract. Potentially eligible studies were retrieved and thoroughly reviewed independently by two reviewers (R.S. and S.E.I.). Those studies that met the selection criteria were included in the review. Differences were resolved by discussion between the two reviewers. The two reviewers (R.S. and S.E.I.) then extracted data from selected studies to be analyzed with Review Manager 5.4 software.

### 2.5. Risk of Bias

Two review authors (R.S. and S.E.I.) independently assessed the risk of bias of each included study. Disagreements were resolved by discussion or consultation with one of the other authors. To assess the risk of bias, we used the criteria recommended by the Cochrane Collaboration [20]: random sequence generation, allocation concealment, blinding of participants and personnel, blinding of outcome assessment, incomplete outcome data, selective reporting and other (analysis for intention to treat and compliance). Each of these criteria was classified as low risk, high risk or uncertain risk of bias. Regarding the blinding of outcome assessment, we considered it a low risk only if the trial described how the outcome measurement was concealed. If it was only stated in the article that this was a double-blind RCT, we considered it an uncertain risk. Regarding the incomplete outcome data, we considered the trial to be of low risk only if the losses were less than 15%; between 15% and 20%, the trial was considered to be of low uncertain risk; and for over 20% of losses, the trial was considered to have a high risk of bias.

### 2.6. Statistical Analyses

Statistical analyses were performed using Review Manager 5.4 software. Considering that all the outcomes analyzed in our review are dichotomous, we calculated the risk ratios (RR) and the 95% confidence intervals (95% CI) for each outcome. For trials with more than two arms, we combined groups to make a single pairwise comparison. As far as possible, we tried to include all participants who were randomized in the denominator of each analysis, and each participant was analyzed in the group to which they were assigned regardless of whether or not they received the intervention, thus following the intention-to-treat principle. Heterogeneity was assessed using the chi² and I² statistics. We considered heterogeneity as important if I² was greater than 30% or the value of *p* associated with chi² was <0.05. If the heterogeneity was significant, the meta-analyses were performed with random effect models; otherwise, we used fixed-effect models. When the meta-analyses included ten or more trials, we investigated publication biases using funnel plots, visually assessing the skewness of the plots. We performed sensitivity analyses to explore the effect of trial quality on different outcomes. The sensitivity analysis was performed, including those articles rated with low risk of bias for the following domains: sequence generation and concealment of allocation, and inadequate blinding.

## 3. Results

The search for trials was carried out during June 2020. Three electronic databases were reviewed: in Central, 574 potentially eligible articles were found; 909 articles were found in Embase; and 1224 articles were found in PubMed Medline. The search strategies are summarized in Table S1, Supplementary Materials. Four articles emerged from the search carried out in relevant journals, conference proceedings and reference lists.

**Figure 1:**
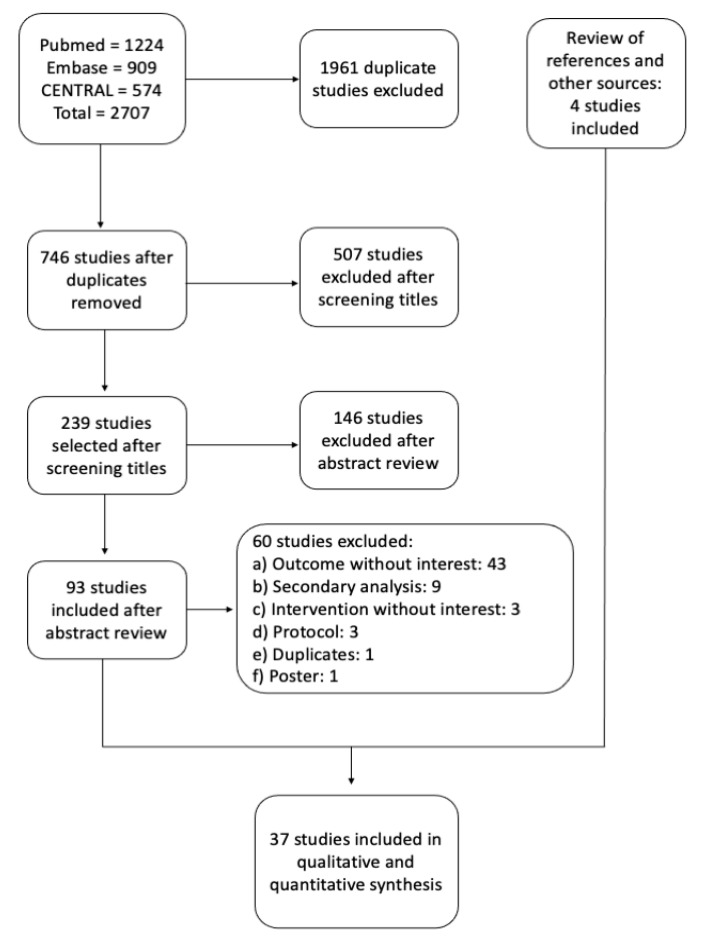
Flow chart of the selection process of randomized controlled trials included in this meta-analysis.

After deleting duplicate articles and screening them for inclusion in the current review based on title and abstract, 97 articles were obtained, which were extensively reviewed. Finally, only 37 articles met the selection criteria and were incorporated into this review. Among the exclusion criteria were the outcomes analyzed that did not relate with the research question, incomplete or inadequate protocols and secondary analysis. The selection process of the included trials is summarized in Figure 1**,** and their characteristics in Table S2, Supplementary Materials.

### 3.1. Main Outcome: Preterm Birth Less Than 37 Weeks

**Figure 2:**
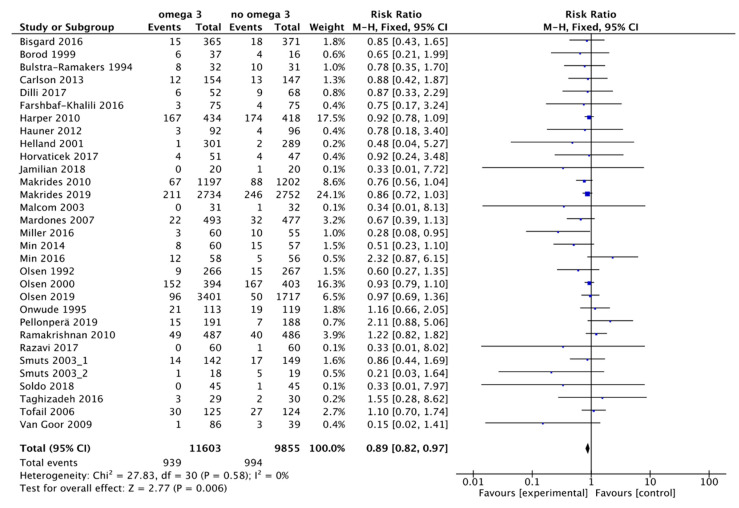
Forest plot of comparison: omega 3 vs. no omega 3, outcome: preterm birth < 37 weeks of gestation.

**Figure 3:**
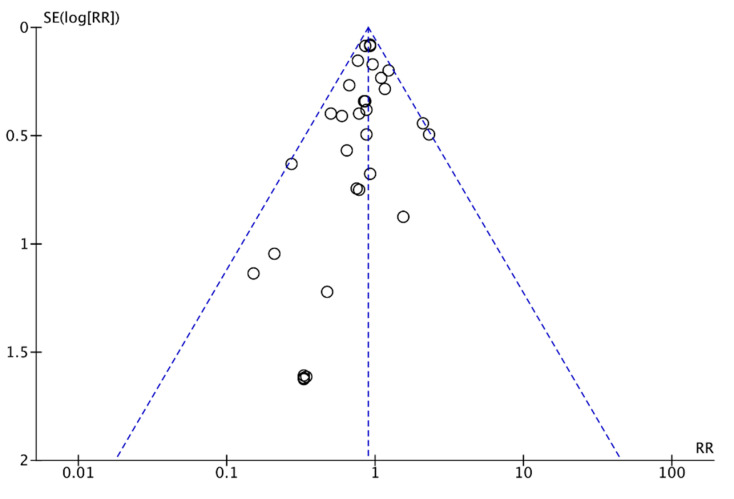
Funnel plot of comparison: omega 3 vs. no omega 3, outcome: preterm birth < 37 weeks of gestation.

In our review, 31 trials were found in which the effect of omega 3 supplements versus placebo or versus compounds without omega 3 was compared to the rate of PTB. The review included 21,458 patients: the experimental group included 11,603 participants, of whom 939 had a PTB, and the control group included 9855 participants, of whom 994 had a PTB (Figure 2). According to these results, there was an 11% reduction in the risk of PTB (RR, 0.89; 95% CI, 0.82 to 0.97). Some asymmetry was observed in the funnel plot’s visual evaluation for this outcome, suggesting the absence of small studies with negative results (Figure 3). Heterogeneity was not significant (I² = 0% *p* associated with chi² = 0.58).

### 3.2. Secondary Outcomes

#### 3.2.1. Early Preterm Delivery

**Figure 4:**
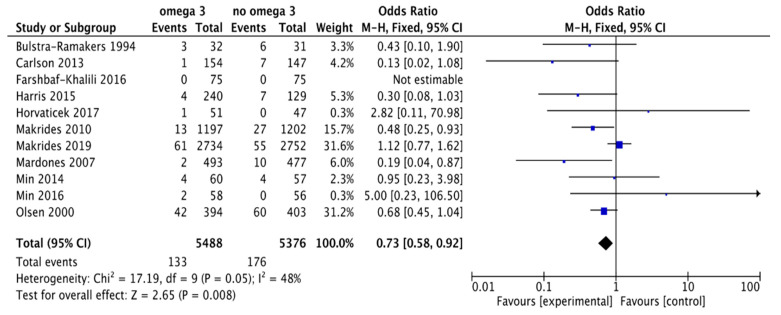
Forest plot of comparison: omega 3 vs. no omega 3, outcome: early preterm birth < 34 weeks of gestation.

**Figure 5:**
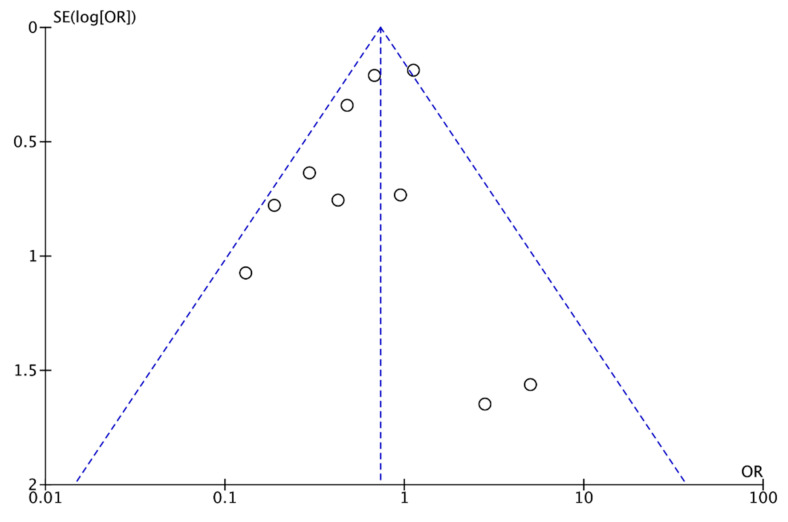
Funnel plot of comparison: omega 3 vs. no omega 3, outcome: early preterm birth < 34 weeks of gestation.

We found 11 trials comparing the effect of omega 3 supplements versus placebo or compounds without omega 3 on the rate of early PTB (ePTB). The review included 10,864 patients: the experimental group included 5488 participants, of whom 133 had ePTB, and the control group included 5376 participants, of whom we found that 176 had ePTB (Figure 4). According to these results, there was a 27% reduction in the risk of ePTB (RR, 0.73; 95% CI, 0.58 to 0.92). Some asymmetry was observed in the funnel plot’s visual evaluation for this outcome, suggesting the absence of some studies with negative results (Figure 5). Heterogeneity was significant (I² = 48% *p* associated with chi² = 0.05).

#### 3.2.2. Preeclampsia or Pregnancy-Induced Hypertension

We found 19 trials that compared the effect of omega 3 supplements versus placebo or versus compounds without omega 3 on the rate of preeclampsia or pregnancy-induced hypertension (PIH). The review included 11,219 patients: the experimental group included 5643 participants, of whom 391 had preeclampsia or PIH, and the control group included 5576 participants, of whom we found that 390 had preeclampsia or PIH (Figure S1, Supplementary Materials). According to these results, there were no differences in the risk of preeclampsia or PIH (RR, 1.00; 95% CI, 0.87 to 1.16). No asymmetry was observed in the funnel plot’s visual evaluation for this outcome (Figure S2, Supplementary Materials). Heterogeneity was not significant (I² = 0%, *p* = 0.68).

#### 3.2.3. Intrauterine Growth Restriction (IUGR)

We found nine trials that compared the effect of omega 3 supplements versus placebo or versus compounds without omega 3 on the IUGR rate. The review included 10,467 patients: the experimental group included 5237 participants, of whom 595 had IUGR, and the control group included 5230 participants, of whom we found that 575 had IUGR (Figure S3, Supplementary Materials). According to these results, there were no differences in the risk of IUGR (RR, 1.04; 95% CI, 0.92 to 1.18). Heterogeneity was not significant (I² = 0%, *p* = 0.76).

#### 3.2.4. Fetal Death

We found 17 trials that compared the effect of omega 3 supplements versus placebo or versus compounds without omega 3 on the rate of intrauterine fetal death (IUFD). The review included 17,517 patients: the experimental group included 9647 participants, 74 with IUFD, and 7870 control participants, of whom we found 54 with IUFD (Figure S4, Supplementary Materials). According to these results, there were no differences in the risk of IUFD (RR, 1.20; 95% CI, 0.85 to 1.69). No asymmetry was observed in the funnel plot’s visual evaluation for this outcome (Figure S5, Supplementary Materials). Heterogeneity was not significant (I² = 0%, *p* = 0.93).

#### 3.2.5. Neonatal Death

We identified ten trials that compared the effect of omega 3 supplements versus placebo or versus compounds without omega 3 on the neonatal death rate. The review included a total of 16,066 patients: the experimental group included 8921 participants, of whom 30 experienced neonatal death, and the control group included 7145 participants, of whom 32 experienced neonatal death (Figure S6, Supplementary Materials). According to these results, there were no differences in neonatal death risk (RR, 0.81; 95% CI, 0.50 to 1.34). No asymmetry was observed in the funnel plot’s visual evaluation for this outcome (Figure S7, Supplementary Materials). Heterogeneity was not significant (I² = 0%, *p* = 0.79).

### 3.3. Sensitivity Analysis

**Figure 6:**
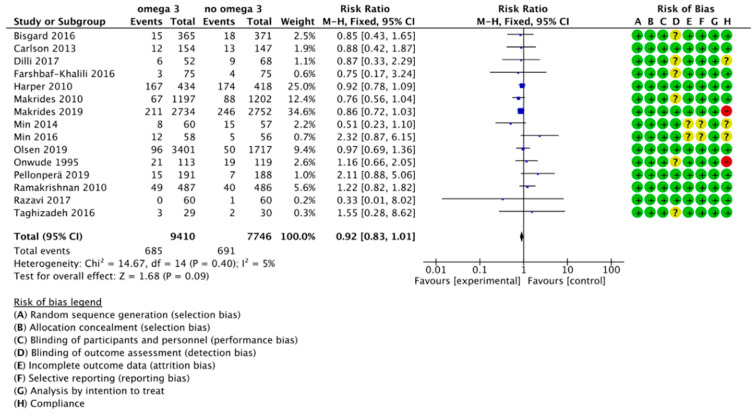
Forest plot of comparison: Sensitivity analysis, outcome: preterm birth (<37 weeks of gestation).

**Figure 7:**
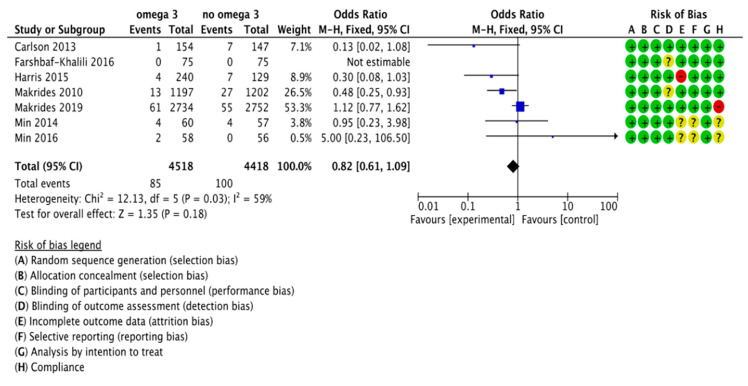
Forest plot of comparison: Sensitivity analysis, outcome: early preterm birth (< 34 weeks of gestation).

We performed sensitivity analyses to explore the effects of trial quality, including only articles considered low risk for random sequence generation, allocation concealment and blinding of participants and personnel. We restricted the sensitivity analysis to the outcome of PTB (main outcome, 15 out of 37 studies) and ePTB (7 out of 11 studies). According to this analysis, there were no significant differences in PTB risk between the group supplemented with omega 3 and the control group (RR, 0.92; 95% CI, 0.83 to 1.01). The results of the analysis are summarized in Figure 6. We also did not find statistically significant differences for the risk of ePTB (RR, 0.82; 95% CI, 0.61 to 1.09). The results are summarized in Figure 7.

### 3.4. Risk of Bias

The risk of bias of the included trials is summarized in Figures S8 and S9 in the Supplementary Materials, which includes random sequence generation, allocation concealment, blinding of participants and personnel, blinding of outcome assessment, incomplete outcome data and selective reporting. In addition to the risks of bias recommended by Higgins 2011 [20], we evaluated two possible biases that could affect the validity of the articles: intention-to-treat and compliance. We considered that the intention-to-treat principle was violated if there was post-randomization exclusion of participants. We considered compliance low risk if it was measured and was greater than 80%.

## 4. Discussion

Preterm delivery is a leading cause of neonatal and childhood mortality and is associated with cognitive deficiencies and increased risk of cardio-metabolic diseases in adult life [21,22]. LC-PUFA are involved in optimum fetoplacental growth and development through their function associated with oxidative stress, angiogenesis and inflammation [23]. The consumption of oily fish and DHA supplements during pregnancy has been associated with maternal and infant benefits, including decreased maternal blood pressure [24] and infant neurodevelopment and growth [25]. Previous studies have explored the relationship between omega 3 intake during pregnancy and timing of delivery or preterm risk; however, the results are still inconsistent. Meanwhile, some clinical trials reported an increase in gestational length after DHA and fish oil supplement interventions [26], and others reported no effect in the incidence of ePTB [27] and PTB [15]. A previous meta-analysis also analyzed the effect of omega 3 supplementation on PTB rates; in 2015, Saccone and Berghella reported no effect of omega 3 with only two RCTs analyzed [28]. However, in 2016, Kar et al., following the analyses of nine studies, did find a positive effect of omega 3 supplementation on the prevention of PTB [29]. Besides including a greater number of studies, compared with Kar and Colleagues, our study provides an update of RCTs published until June 2020 as well as the analyses of secondary outcomes, such as preeclampsia, IUGR and fetal and neonatal death.

Based on the present meta-analysis results, we conclude that omega 3 supplementation during pregnancy does not reduce the risk of PTB and ePTB. Although preliminary results showed that omega 3 supplementation reduced the risk of PTB and ePTB by 11% and 27%, respectively, after sensitivity analyses were performed and only low risk of bias studies were analyzed, the significance of the effect of omega 3 on PTB and ePTB risk disappears. Concerning the effect of omega 3 supplementation on other perinatal outcomes, such as preeclampsia, IUGR risk and fetal and neonatal death, no differences were found in this study. These results suggest that among the studies analyzed, there is not enough evidence suggesting that omega 3 supplementation during pregnancy decreases the risk of PTB and other perinatal complications.

The form of omega 3 supplementation in the analyzed trials differed and included capsules; liquid fish oil; and enriched food products, such as eggs high in DHA and bars containing DHA. The intake of 600 mg of DHA/day as capsules or bars has shown an increased length of pregnancy by 2.9 days [12] and 4.0–4.5 days [30]; meanwhile, the ingestion of 137 mg of DHA from high-DHA eggs showed a gestation increase of 6 days [26]. It can be hypothesized that food-based DHA (i.e., DHA-rich eggs) might have a more pronounced impact on pregnancy lengths as omega 3 bioavailability is higher when DHA is consumed in a high-fat food matrix [31]. The administration of omega 3 as DHA and EPA by itself or in combination with other nutrients should also be considered at the moment of describing the effect of omega 3 on fetal and maternal outcomes. In this review, considering the number of studies, it was not possible to differentiate between different doses and different components of supplementations. Similarly, nutritional status and omega 3 deficiency should also be considered. Lower levels of plasma EPA and DHA showed a 10-fold increased risk of ePTB compared to the higher plasma levels [8], demonstrating a potential benefit of the supplementation effect during deficiency.

Timing and length of supplementation are important factors determining the effect of omega 3 on preterm delivery risk. Considering the mechanism of action of omega 3 associated with inflammation and electrical activity of myometrium, it can be expected that acute (short-term) and chronic (throughout the pregnancy) supplementation have different effects on outcomes related to preterm delivery. The time of administration, the gestational week when the supplementation started and if the treatment continued through the whole pregnancy or not are characteristics that have to be considered in future studies. The potential adverse effects of omega 3 supplementation are not discussed in this review; however, post-term partition or bleeding have been associated with high omega 3 doses (>2.7 g/day) [32].

Furthermore, the strengths and limitations of this study should be considered. Our study’s strength is that 37 studies were analyzed, which included a total of 21,458 and 10,864 women for PTB and ePTB risk analyses, respectively. This studies are summarized in Table S2 [10,11,12,13,14,15,24,26,27,30,33,34,35,36,37,38,39,40,41,42,43,44,45,46,47,48,49,50,51,52,53,54,55,56,57,58,59]. Although risk of bias was considered in the study, additionally, a sensitivity analysis was performed to assess each publication’s reliability. There are, however, some limitations to this study. Firstly, among the studies that met the inclusion criteria and were considered low risk of bias, only 15/31 and 7/11 trials for PTB and ePTB studies, respectively, were included after sensitivity analyses. This selection removed the significant results that were found early in the study. Therefore, more RCTs with high-quality standards may be required to increase the number of RCTs included in the sensitivity analyses. Secondly, the oldest trial analyzed was published in 1992; this may suggest differences in the baseline nutritional status and diet style of the participants compared with the newest RCTs and could generate a higher heterogenicity among demographic and anthropometric characteristics of women. Finally, the type of dose, timing and length of supplementation were not considered in the analysis, which are all aspects that can influence the effect of omega 3 supplementation on PTB prevention.

## 5. Conclusions

In summary, our results suggest that there is not enough available evidence that supports the conclusion that omega 3 supplementation during pregnancy reduces the risk of PTB and ePTB. However, more research is needed to identify the impact of fish oil and omega 3 supplementation on PTB rates.

## Data Availability

Not applicable.

## Supplementary Materials

The following are available online at https://www.mdpi.com/article/10.3390/nu13051704/s1, Table S1: Keywords and search strategies; Table S2: Characteristics of randomized controlled trials (RCTs) included in systematic review; Figure S1 Forest plot of comparison: omega 3 vs. No omega 3, outcome: preeclampsia or pregnancy-induced hypertension (PIH); Figure S2: Funnel plot of comparison: omega 3 vs. no omega 3, outcome: preeclampsia or pregnancy-induced hypertension (PIH); Figure S3: Forest plot of comparison: omega 3 vs. no omega 3, outcome: IUGR (<p10); Figure S4: Forest plot of comparison: omega 3 vs. no omega 3, outcome: fetal death; Figure S5: Funnel plot of comparison: omega 3 vs. no omega 3, outcome: fetal death; Figure S6: Forest plot of comparison: omega 3 vs. no omega 3, outcome: neonatal death; Figure S7: Funnel plot of comparison: omega 3 vs. no omega 3, outcome: neonatal death; Figure S8: Risk of bias graph; and Figure S9: Risk of bias summary.
