# Supplementary material for: Supplementation of Omega 3 during Pregnancy and the Risk of Preterm Birth: A Systematic Review and Meta-Analysis
Source: Nutrients. 2021 May 18;13(5):1704. doi: 10.3390/nu13051704 (PMC8157397; doi:10.3390/nu13051704)
Supplement: Supplementary file 1 [file nutrients-13-01704-s001.zip › Figures Supplementary Materials Nutrients.pdf]

CENTRAL:	
#1	MeSH descriptor: [Pregnancy] explode all trees
#2	MeSH descriptor: [Fatty Acids, Omega-3] explode all trees
#3	#1 AND #2 in Trials (77)
#4	("pregnancy"):kw AND ("omega 3"):kw in Trials (Word variations have been searched) (161)
#5	("pregnancy"):kw AND ("DHA"):kw in Trials (Word variations have been searched) (0)
#6	("pregnancy"):kw AND ("fish oil"):kw in Trials (Word variations have been searched) (101)
#7	("pregnancy"):kw AND ("eicosapentaenoic acid"):kw in Trials (Word variations have been searched) (28)
#8	("pregnancy"):kw AND ("docosahexaenoic acid"):kw in Trials (Word variations have been searched) (183)
#9	("pregnancy"):kw AND ("linoleic acid"):kw in Trials (Word variations have been searched) (24)
EMBASE:	
#1	'pregnancy'/exp AND 'omega 3 fatty acid'/exp AND 'clinical trial'/exp: 199
#2	'pregnancy'/exp AND dha AND 'clinical trial'/exp: 174
#3	'pregnancy'/exp AND n3 AND 'clinical trial'/exp: 113
#4	'pregnancy'/exp AND 'fish oil'/exp AND 'clinical trial'/exp: 137
#5	'pregnancy'/exp AND 'eicosapentaenoic acid'/exp AND 'clinical trial'/exp: 75
#6	'pregnancy'/exp AND 'docosahexaenoic acid'/exp AND 'clinical trial'/exp: 170
#7	'pregnancy'/exp AND 'linoleic acid'/exp AND 'clinical trial'/exp: 41
PUBMED MEDLINE:<br>Search: "Pregnancy"[Mesh] AND linoleic acid Filters: Clinical Trial 56	
Search: "Pregnancy"[Mesh] AND docosahexaenoic acid Filters: Clinical Trial 190	
Search: "Pregnancy"[Mesh] AND eicosapentaenoic acid Filters: Clinical Trial 85	
Search: ("Pregnancy"[Mesh]) AND "Fish Oils"[Mesh] Filters: Clinical Trial 270	
Search: "Pregnancy"[Mesh] AND n3 Filters: Clinical Trial 210	
Search: "Pregnancy"[Mesh] AND dha Filters: Clinical Trial 166	
Search: ("Pregnancy"[Mesh]) AND "Fatty Acids, Omega-3"[Mesh]Filters: Clinical Trial 247	

Table S1: Keywords and search strategies.

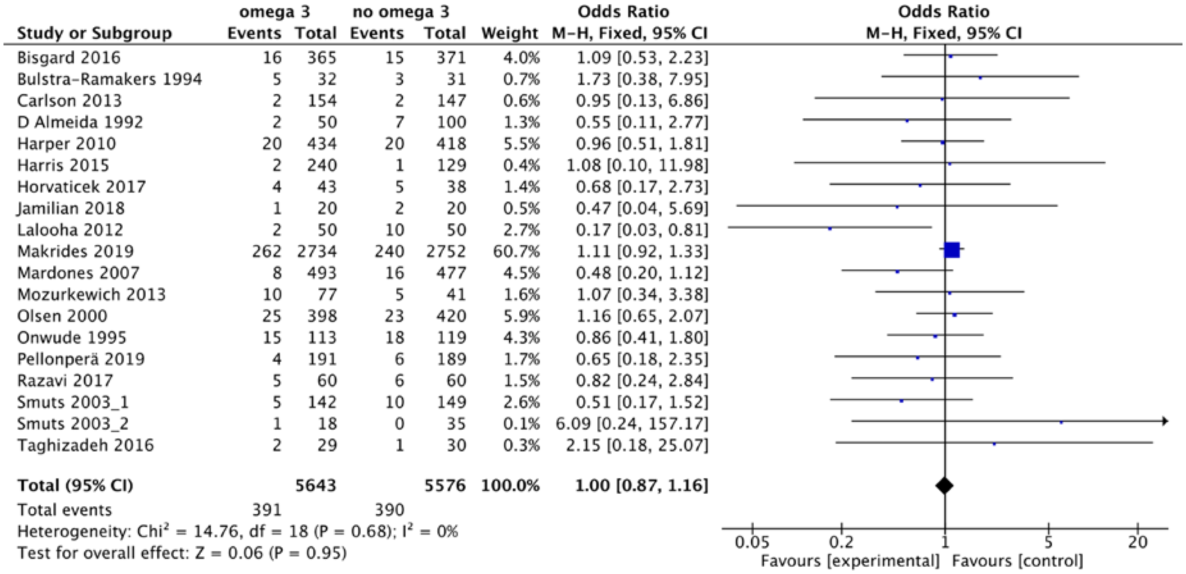

Study or Subgroup	omega 3 Events	omega 3 Total	no omega 3 Events	no omega 3 Total	Weight	Odds Ratio M-H, Fixed, 95% CI
Bisgard 2016	16	365	15	371	4.0%	1.09 [0.53, 2.23]
Bulstra-Ramakers 1994	5	32	3	31	0.7%	1.73 [0.38, 7.95]
Carlson 2013	2	154	2	147	0.6%	0.95 [0.13, 6.86]
D Almeida 1992	2	50	7	100	1.3%	0.55 [0.11, 2.77]
Harper 2010	20	434	20	418	5.5%	0.96 [0.51, 1.81]
Harris 2015	2	240	1	129	0.4%	1.08 [0.10, 11.98]
Horvaticek 2017	4	43	5	38	1.4%	0.68 [0.17, 2.73]
Jamilian 2018	1	20	2	20	0.5%	0.47 [0.04, 5.69]
Lalooha 2012	2	50	10	50	2.7%	0.17 [0.03, 0.81]
Makrides 2019	262	2734	240	2752	60.7%	1.11 [0.92, 1.33]
Mardones 2007	8	493	16	477	4.5%	0.48 [0.20, 1.12]
Mozurkewich 2013	10	77	5	41	1.6%	1.07 [0.34, 3.38]
Olsen 2000	25	398	23	420	5.9%	1.16 [0.65, 2.07]
Onwude 1995	15	113	18	119	4.3%	0.86 [0.41, 1.80]
Pellonperä 2019	4	191	6	189	1.7%	0.65 [0.18, 2.35]
Razavi 2017	5	60	6	60	1.5%	0.82 [0.24, 2.84]
Smuts 2003_1	5	142	10	149	2.6%	0.51 [0.17, 1.52]
Smuts 2003_2	1	18	0	35	0.1%	6.09 [0.24, 157.17]
Taghizadeh 2016	2	29	1	30	0.3%	2.15 [0.18, 25.07]
**Total (95% CI)**		**5643**		**5576**	**100.0%**	**1.00 [0.87, 1.16]**
Total events	391		390			

Heterogeneity: $Chi^2 = 14.76$, df = 18 (P = 0.68); $I^2 = 0\%$
Test for overall effect: Z = 0.06 (P = 0.95)

Figure S1. Forest plot of comparison: Omega 3 vs No Omega 3, outcome: preeclampsia or pregnancy-induced hypertension (PIH).

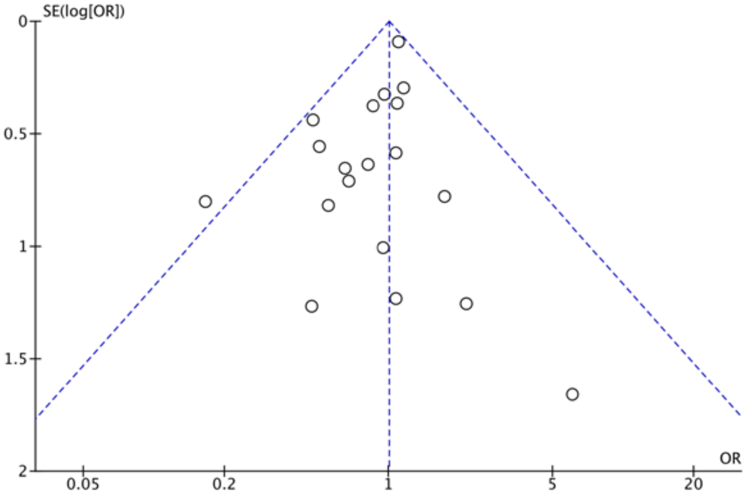

Figure S2. Funnel plot of comparison: Omega 3 vs No Omega 3, outcome: preeclampsia or pregnancy-induced hypertension (PIH).

Study or Subgroup	omega 3 Events	omega 3 Total	no omega 3 Events	no omega 3 Total	Weight	Odds Ratio M-H, Fixed, 95% CI
Bulstra-Ramakers 1994	11	32	9	31	1.3%	1.28 [0.44, 3.71]
Harper 2010	35	427	41	410	8.0%	0.80 [0.50, 1.29]
Makrides 2019	206	2787	196	2800	37.9%	1.06 [0.87, 1.30]
Mardones 2007	30	493	39	477	7.8%	0.73 [0.44, 1.19]
Olsen 2000	208	685	185	689	26.9%	1.19 [0.94, 1.50]
Onwude 1995	33	113	35	119	5.1%	0.99 [0.56, 1.74]
Pellonperä 2019	15	184	16	188	3.0%	0.95 [0.46, 1.99]
Ramakrishnan 2010	55	487	53	486	9.8%	1.04 [0.70, 1.55]
Taghizadeh 2016	2	29	1	30	0.2%	2.15 [0.18, 25.07]
**Total (95% CI)**		**5237**		**5230**	**100.0%**	**1.04 [0.92, 1.18]**
Total events	595		575			

Heterogeneity: $Chi^2 = 4.99$, $df = 8$ ($P = 0.76$); $I^2 = 0\%$
Test for overall effect: $Z = 0.68$ ($P = 0.50$)

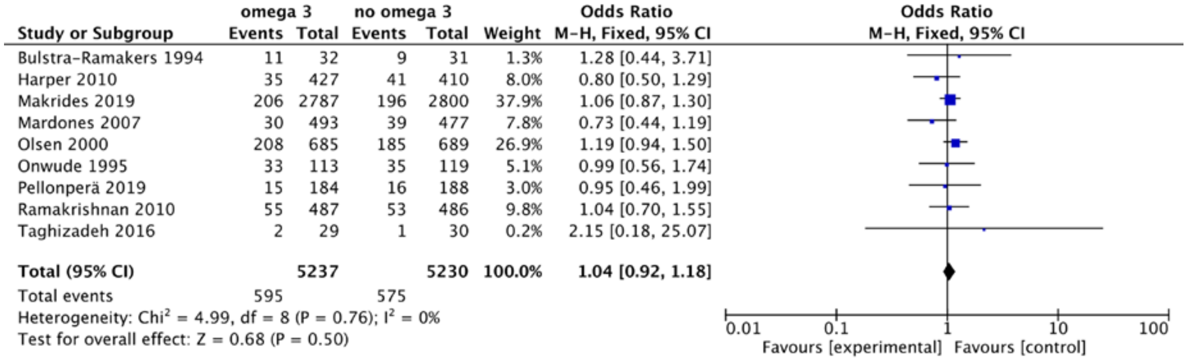

Figure S3. Forest plot of comparison: Omega 3 vs No Omega 3, outcome: IUGR (< P10).

Study or Subgroup	omega 3 Events	omega 3 Total	no omega 3 Events	no omega 3 Total	Weight	Odds Ratio M-H, Fixed, 95% CI
Bisgard 2016	2	365	2	371	3.3%	1.02 [0.14, 7.26]
Bulstra-Ramakers 1994	1	32	0	31	0.8%	3.00 [0.12, 76.49]
De Groot 2004	0	40	1	39	2.5%	0.32 [0.01, 8.02]
Haghiac 2015	1	25	0	25	0.8%	3.12 [0.12, 80.39]
Helland 2001	1	301	0	289	0.9%	2.89 [0.12, 71.24]
Horvaticek 2017	1	52	0	50	0.8%	2.94 [0.12, 73.93]
Makrides 2019	16	2734	13	2752	21.7%	1.24 [0.60, 2.58]
Min 2014	2	60	0	57	0.8%	4.91 [0.23, 104.62]
Min 2016	0	58	1	57	2.5%	0.32 [0.01, 8.07]
Olsen 1992	1	266	1	267	1.7%	1.00 [0.06, 16.13]
Olsen 2000	16	1056	19	1085	31.1%	0.86 [0.44, 1.69]
Olsen 2019	22	3676	5	1855	11.1%	2.23 [0.84, 5.89]
Onwude 1995	0	113	2	119	4.1%	0.21 [0.01, 4.36]
Pellonperä 2019	0	191	1	189	2.5%	0.33 [0.01, 8.11]
Ramakrishnan 2010	2	489	3	489	5.0%	0.67 [0.11, 4.00]
Taghizadeh 2016	1	30	0	30	0.8%	3.10 [0.12, 79.23]
Tofail 2006	8	159	6	165	9.4%	1.40 [0.48, 4.14]
**Total (95% CI)**		**9647**		**7870**	**100.0%**	**1.20 [0.85, 1.69]**
Total events	74		54			

Heterogeneity: $Chi^2 = 8.60$, $df = 16$ ($P = 0.93$); $I^2 = 0\%$
Test for overall effect: $Z = 1.03$ ($P = 0.30$)

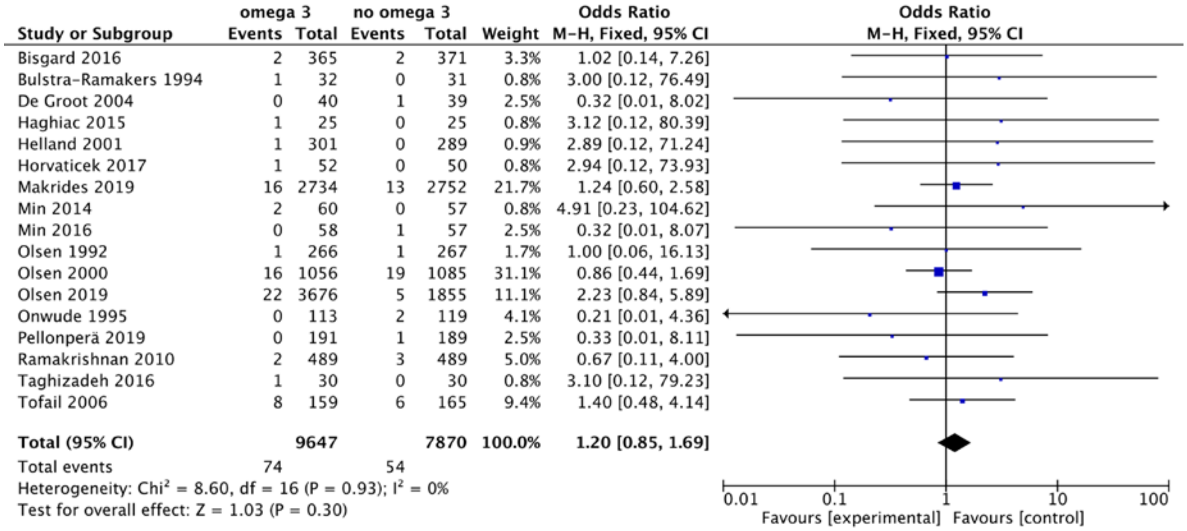

Figure S4. Forest plot of comparison: Omega 3 vs No Omega 3, outcome: fetal death.

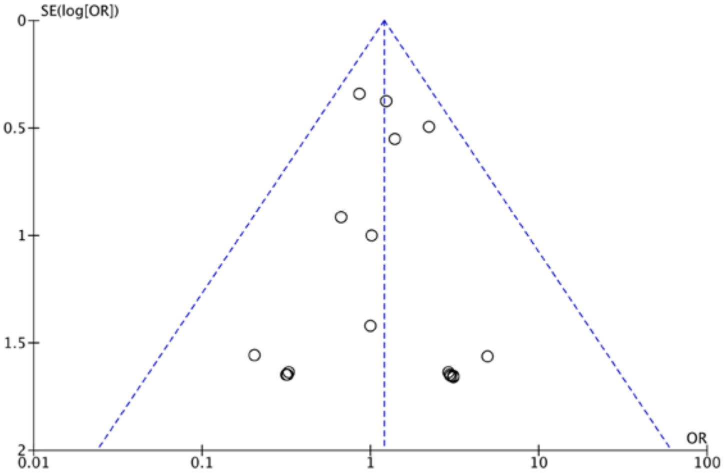

Figure S5: Funnel plot of comparison: 1 Omega 3 vs No Omega 3, outcome: fetal death.

Study or Subgroup	omega 3 Events	omega 3 Total	no omega 3 Events	no omega 3 Total	Weight	Odds Ratio M-H, Fixed, 95% CI
Bisgard 2016	0	365	0	371		Not estimable
Bulstra-Ramakers 1994	1	32	3	31	8.5%	0.30 [0.03, 3.06]
Carlson 2013	1	154	1	147	2.9%	0.95 [0.06, 15.40]
Farshbaf-Khalili 2016	0	75	1	75	4.3%	0.33 [0.01, 8.20]
Makrides 2019	8	2734	4	2752	11.5%	2.02 [0.61, 6.70]
Olsen 2000	3	1126	4	1144	11.5%	0.76 [0.17, 3.41]
Olsen 2019	8	3676	6	1855	23.0%	0.67 [0.23, 1.94]
Onwude 1995	1	113	0	119	1.4%	3.19 [0.13, 79.04]
Ramakrishnan 2010	4	487	8	486	23.0%	0.49 [0.15, 1.65]
Tofail 2006	4	159	5	165	13.8%	0.83 [0.22, 3.13]
**Total (95% CI)**		**8921**		**7145**	**100.0%**	**0.81 [0.50, 1.34]**
Total events	30		32			

Heterogeneity: $Chi^2 = 4.69$, df = 8 (P = 0.79); $I^2 = 0\%$
Test for overall effect: Z = 0.81 (P = 0.42)

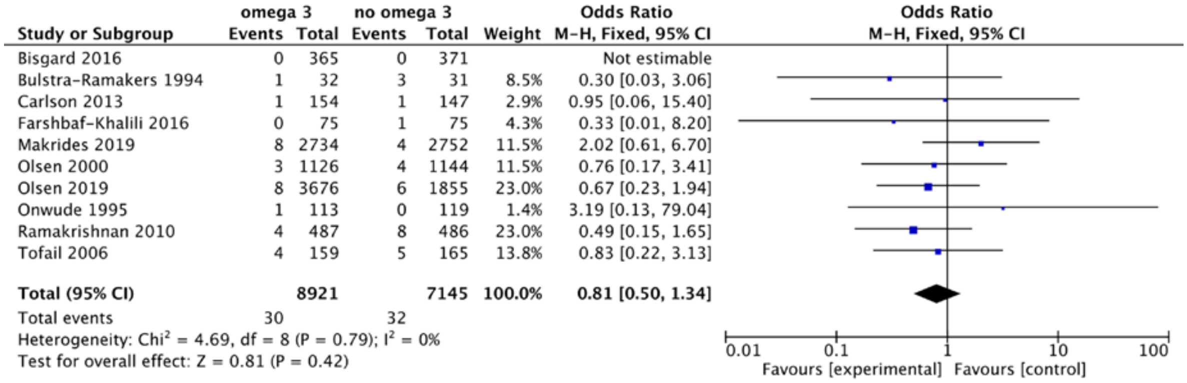

Figure S6. Forest plot of comparison: 1 Omega 3 vs No Omega 3, outcome: neonatal death.

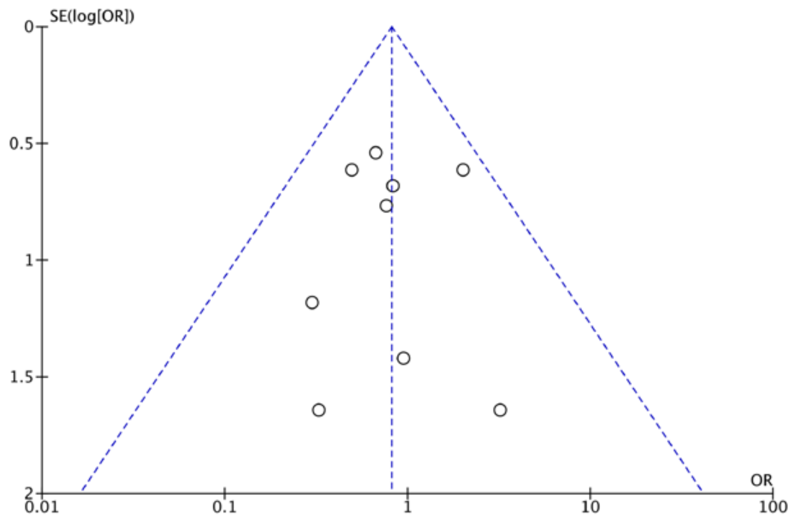

Figure S7. Funnel plot of comparison: 1 Omega 3 vs No Omega 3, outcome: neonatal death.

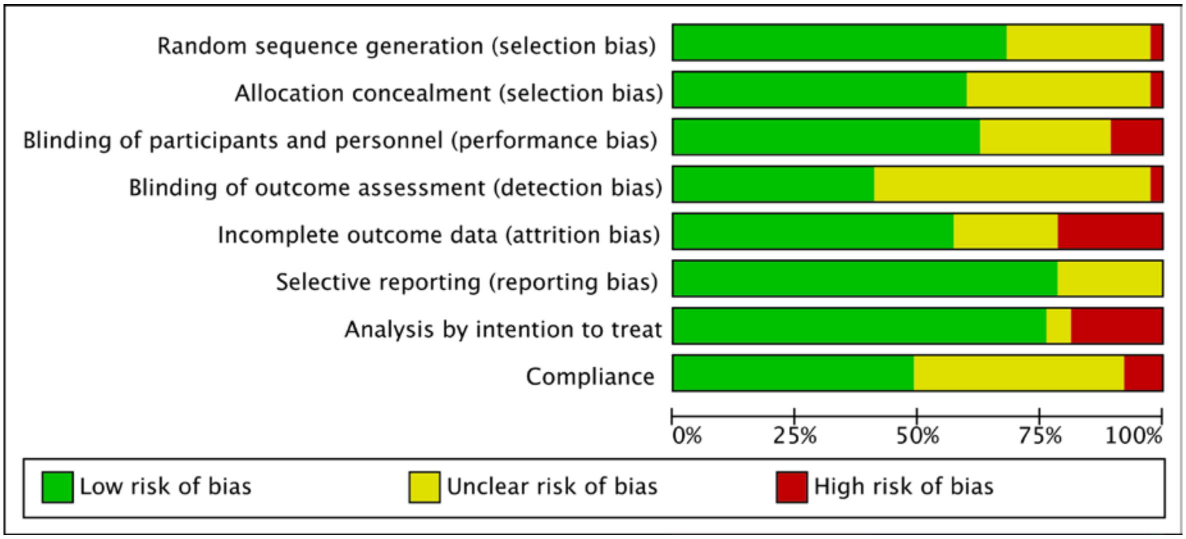

Figure S8. Risk of bias graph: review authors' judgements about each risk of bias item presented as percentages across all included studies.

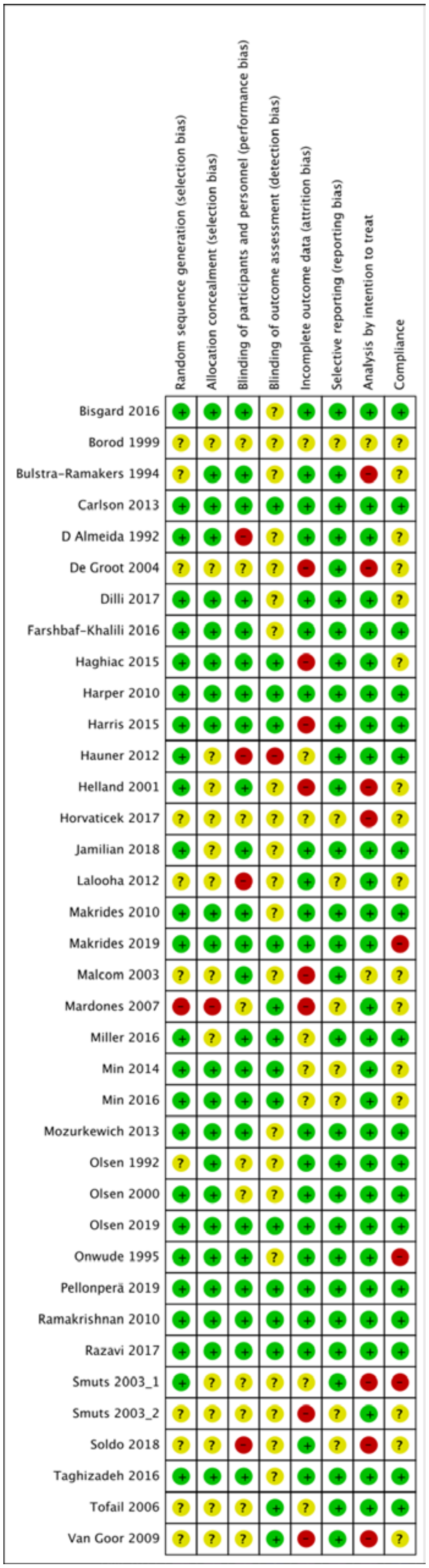

Figure S9. Risk of bias summary: review authors' judgements about each risk of bias item for each included study.
