# Supplementary material for: Supplementation of Omega 3 during Pregnancy and the Risk of Preterm Birth: A Systematic Review and Meta-Analysis
Source: Nutrients. 2021 May 18;13(5):1704. doi: 10.3390/nu13051704 (PMC8157397; doi:10.3390/nu13051704)
Supplement: Supplementary file 1 [file nutrients-13-01704-s001.zip › Table S2 RCT RF 13.05.pdf]

Table S2. Characteristics of randomized controlled trials (RCTs) included in systematic review.

Study	Methods	Objective	Setting	Interventions	Timing of supplementation	Outcome	DHA + EPA dose/day
**Jamilian 2018 [33]**	RCT, double-blinded placebo-controlled, 2 arms	To determine the effect of fish oil administration on gene expression related to insulin action, blood lipids, and inflammation in women with GDM.	Arak University of Medical Sciences, Iran	Participants with GDM (n = 40), aged 18–40 years, were randomized to take either 1000 mg fish oil capsules, containing 180 mg EPA and 120 mg DHA (n = 20), or placebo (n = 20) twice a day for 6 weeks.<br>All 40 women completed the study.	6 weeks	Preterm delivery <37 weeks, preeclampsia	Mid, 240 mg DHA + 360 mg EPA
**Bisgaard 2016 [34]**	RCT, double-blinded placebo-controlled, 2 arms	To assess the effect of supplementation with omega 3 LCPUFAs in healthy pregnant women on the risk of persistent wheeze and asthma in their offspring.	Copenhagen, Denmark	736 women were randomized to: Group 1: 2.4 g per day of fish oil containing 55% EPA and 37% DHA (365 women) or Group 2: placebo containing olive oil (n=371 women).<br>695 participants were included in the analysis (349 in the control group and 346 in the experimental group).	From 24 weeks until 1 week post-delivery.	Preterm delivery <37 weeks, preeclampsia, fetal death, neonatal death.	High, 888 mg DHA + 1320 mg EPA
**Borod 1999 [11]**	RCT, 3 arms	To assess the effect of supplementation with eggs rich in DHA in healthy pregnant women on the maternal DHA levels in the third trimester of gestation and DHA levels in the newborn.	Not reported	53 women were randomized to:<br>Group 1: consume up to one dozen regular eggs, each containing about 28 mg DHA, per week (regular egg)<br>Group 2: consume up to one dozen eggs from chickens fed a diet containing the dried microalgae, each containing approximately 135 mg DHA per week (high-DHA egg);<br>Group 3: routinely consumed few if any eggs (low egg).	From 24 weeks until delivery.	Preterm delivery <37 weeks	Low, 28 to 135 mg DHA

**Bulstra-Ramakers 1994 [35]**	RCT, double-blinded placebo-controlled multicenter, 2 arms	To assess the effect of the supplementation of 3g EPA per day on the recurrence rate of pregnancy induced hypertension and fetal growth retardation in women with a previous history of intrauterine growth retardation (IUGR), with or without pregnancy induced hypertension.	University Hospital and regional hospitals in the north of the Netherlands.	68 women were randomized to mixture of EPA and DHA capsules (containing 3 g of EPA per day) or placebo capsules (contained coconut oil). 63 women were included in the analysis, 32 in EPA group and 31 in the placebo group.	From 12 to 14 weeks until delivery.	Preterm delivery <37 weeks, preterm <34 weeks, preeclampsia, fetal growth restriction, fetal death, neonatal death.	High, DHA not reported + 3000 mg EPA
**Carlson 2013 [12]**	RCT, double-blinded placebo-controlled multicenter, 2 arms	To test the hypothesis that 600 mg/d of the omega 3 LCPUFA DHA can increase maternal and newborn DHA status, gestation duration, birth weight, and length in healthy pregnant women.	Kansas City metropolitan area	350 women were randomized to receive 3 capsules/d of a marine algae-oil source of DHA (200 mg DHA/capsule) or 3 capsules of placebo containing half soybean and half corn oil. 301 women were included in the analysis, 154 in DHA group and 147 in the placebo group.	From 8 to 20 weeks until delivery.	Preterm delivery <37 weeks, preterm delivery <34 weeks, preeclampsia, neonatal death.	Mid, 600 mg DHA + 0 mg EPA
**D Almeida 1992 [36]**	RCT, placebo-controlled, 3 arms	To assess the effect of a combination of primrose oil and fish oil (DHA + EPA) versus magnesium oxide versus placebo in preventing preeclampsia in patients primiparous and multiparous that course the first four months of pregnancy.	Central Maternity Hospital for Luanda, Angola	150 women were randomized to 3 groups:<br>Group 1: placebo (olive oil, without vitamin E).<br>Group 2: 8 capsules per day of a mixture of primrose oil (37 mg GLA) and fish oil (18 mg EPA and 10 mg DHA)<br>Group 3: magnesium oxide 1000 mg per day<br>150 participants were included in the analysis. 50 in the placebo group, 50 in the GLA + EPA + DHA group and 50 in the magnesium oxide group.	From the first four months of pregnancy for six months.	Preeclampsia.	Low, 80 mg DHA + 144 mg EPA

**De Groot 2004 [37]**	RCT, double-blinded, 2 arms	To improve maternal and neonatal fatty acid status by supplementing pregnant women with a combination of alfa-linolenic acid (ALA) and linoleic acid (LA), the ultimate dietary precursors of DHA and AA, respectively in healthy pregnant women	Southeastern part of the Netherlands	79 women were randomly assigned to receive daily >= 25 g of either an ALA-enriched, high-LA margarine (experimental group) or a high-LA margarine without ALA (control group). 58 women were included in the analysis, 29 in the control group and 29 in the experimental group.	From week 14 of pregnancy until delivery.	Fetal death.	0 mg DHA + 0 mg EPA, + 2.8 g ALA
**Dilli 2017 [38]**	RCT, multicenter, placebo-controlled, 2 arms	To investigate the potential effects of maternal fish oil supplementation on cord blood IGF1 DNA methylation profiles in women with gestational diabetes mellitus (GDM)	Maternity and children's hospitals from different geographical regions of Turkey	140 women with GDM were randomized to receive 1 capsule/d with EPA 384 mg and DHA 252 mg or 1 capsule of placebo containing sunflower oil. 120 women were included in the analysis, 68 in the placebo group and 52 in the experimental group.	From 24-28 weeks until delivery.	Preterm delivery <37 weeks.	Mid, 252 mg DHA + 384 mg EPA
**Haghiac 2015 [39]**	RCT, double-blinded, placebo-controlled, 2 arms	To characterize the effects of omega 3 fatty acids supplements on the inflammatory status in the placenta and adipose tissue of overweight/obese pregnant women.	MetroHealth Medical Center.	72 women were randomized to receive oral 800 mg DHA and 1200 mg of EPA for a total of 2,000 mg of omega 3 LCPUFAs, divided into 4 capsules, or matching placebo capsules. 49 women were included in the analysis, 24 in the placebo group and 25 in the experimental group.	From 10-16 weeks until delivery.	Fetal death	High, 800 mg DHA + 1200 mg EPA
**Harper 2010 [13]**	RCT, multicenter, placebo-controlled, double-masked, 2 arms	To test the hypothesis that among women with at least one prior spontaneous preterm delivery receiving weekly 17 alfa-hydroxyprogesterone caproate the addition of an omega 3 supplement would further reduce the rate of recurrent preterm birth.	13 centers of prenatal control in USA.	852 women were randomized to receive 1.200 mg EPA and 800 mg DHA per day or matching placebo capsules which contained only a minute amount of inert mineral oil. 852 women were included in the analysis, 418 in the placebo group and 434 in the experimental group.	From 16 - 22 weeks until 36 weeks.	Preterm delivery <37 weeks, fetal growth restriction, preeclampsia.	High, 800 mg DHA + 1200 mg EPA

**Harris 2015 [30]**	RCT, double-blinded, placebo-controlled, 3 arms + 1 arm non randomized	To compare supplementation at two levels of DHA oil to nutrition education targeted to increase DHA consumption from fish and DHA fortified foods on gestational length in healthy pregnant women	Denver Health Hospitals and Clinics, Denver, Colorado, USA	Interventions: 634 women were randomized to 3 groups: Group 1: bars o gel capsules with 300 mg of DHA (200 women). Group 2: bars o gel capsules with 600 mg oh DHA (221 women). Group 3: placebo, bars o gel capsules with olive oil (213 women). 209 women were assigned to nutrition education arm (no randomized) 345 participants were included in the analysis. 121 in the placebo group, 224 in the experimental group (107 in the DHA 300 mg group and 117 in the DHA 600 mg group), and 191 in the nutrition education group.	From 20 weeks until delivery.	Preterm delivery <34 weeks, preeclampsia.	Low - mild, 300 - 600 mg DHA, 0 mg EPA
**Hauner 2012 [40]**	RCT, 2 arms.	To test that a reduction in the omega-6: omega-3 LCPUFA ratio in the diet of healthy pregnant women may prevent expansive adipose tissue growth in their infants during the first year of life.	Division of Obstetrics and Perinatal Medicine of the University Hospital Klinikum rechts der Isar, Technische Universität München, Germany	208 women were randomized to 2 groups: Group 1: fish-oil supplement as capsules that contained 1200 mg omega 3 LCPUFAs (1020 mg DHA and 180 mg EPA) as well as 9 mg vitamin E as an antioxidant per day during pregnancy and lactation (104 women). Group 2: detailed nutritional counseling from trained research assistants (104 women). 188 women were included in the analysis. 96 in the placebo group and 92 in the experimental group.	From 15 weeks until to 4 months of lactation.	Preterm delivery <37 weeks.	High, 1020 mg DHA and 180 mg EPA

**Helland 2001 [41]**	RCT, 2 arms.	To investigate whether dietary supplementation of long-chain omega 3 fatty acids to healthy pregnant women and lactating mothers would affect gestational length, birth weight, and the biochemical status of the neonates	The National Hospital, Oslo, Norway.	590 women were randomized to 2 groups: Group 1: 10 ml per day of cod liver oil containing 1183 mg of DHA + 803 mg of EPA (301 women). Group 2: 10 mL per day of corn oil (289 women).<br>341 women were included in the first analysis (time of delivery). 166 in the control group and 175 in the experimental group.	From 17-19 weeks until to 3 months postpartum.	Preterm delivery <37 weeks, fetal death	High, 1183 mg DHA + 803 mg EPA
**Horvaticek 2017 [42]**	RCT, double-blinded placebo-controlled, 2 arms.	To investigate the effect of pregnancy and EPA and DHA on C-peptide secretion in pregnant women with Type-1 diabetes mellitus	Department of Obstetrics and Gynecology, Zagreb University Hospital Center, Zagreb, Croatia	109 women were randomized to 2 groups: Group 1: 2 capsules for day of EPA + DHA. Each capsule contained EPA 60 mg + DHA 308 mg (47 women). Group 2: Placebo (corn oil), 43 women.<br>90 women were included in the analysis. 43 in the control group and 47 in the experimental group.	From 9 weeks until delivery	Preterm delivery <37, preterm delivery <34, preeclampsia, fetal death	Mid, 616 mg DHA + 120 mg EPA
**Taghizadeh 2016 [43]**	RCT, double-blinded placebo-controlled, 2 arms	To determine the effects of omega 3 fatty acids and vitamin E supplementation on biomarkers of oxidative stress, inflammation and pregnancy outcomes of patients with GDM who were not taking oral hypoglycemic agents.	Kosar Clinic in Arak, Iran	Participants were randomly divided into 2 groups to receive either omega 3 fatty acids plus vitamin E supplements (n=30) or placebo (n=30) for 6 weeks. 60 women were included in the analysis.	From 24 to 28 weeks of gestation for 6 weeks	Outcome: Preterm delivery <37 weeks, preeclampsia, small for gestational age, intrauterine fetal death.	400 mg alpha-linolenic acid plus 400 IU vitamin E per day for 6 weeks
**Farshbaf-Khalili 2017 [44]**	RCT double-blind, placebo-controlled, 2 arms	To determine the average serum level of DHA and EPA in healthy pregnant women with a single pregnancy.	Tabriz- Iran	Participants were randomly divided into 2 groups to receive fish oil 1000 mg per day containing 120 mg of DHA, 180 mg of EPA and 400 mg of ALA (n=75) or placebo (n=75). All women were included in the analysis.	From 20 weeks of gestation until delivery.	Preterm delivery <37 weeks, preterm delivery <34 weeks, neonatal death.	Low, 120mg of DHA, 180mg of EPA.

**Lalooha 2012 [24]**	RCT, single-blind, placebo-controlled, 2 arms	To determine the effect of use of omega-3 on reduction of preeclampsia risk in women with risk of preeclampsia and gestational age of 14 to 18 weeks.	Qazvin city, Iran.	Participants were randomly divided into 2 groups to receive omega 3 supplement 1000 mg per day containing DHA + EPA (n=50) or placebo (n=50). All women were included in the analysis.	From 14-18 weeks of pregnancy until delivery.	Preeclampsia.	Not reported, only described 1000 mg por day of DHA + EPA
**Makrides 2010 [14]**	RCT, double-blind, multicenter, 2 arms	To determine whether DHA supplementation during the last half of pregnancy reduced the risk of depressed maternal mood during the postpartum period and improved early cognitive development in the offspring in healthy pregnant women with singleton pregnancies at less than 21 weeks of gestation.	5 Australian perinatal centers.	Participants were randomly divided into 2 groups to receive DHA–rich fish oil capsules providing 800 mg/d of DHA and 100 mg of EPA (n=1197) or matched vegetable oil capsules without DHA (n=1202).<br>All women were included in the primary analysis	From 14-18 weeks of pregnancy until delivery.	Preterm delivery <37 weeks, preterm delivery <34 weeks.	Mid, 800 mg of DHA, 100 mg of EPA
**Makrides 2019 [27]**	RCT, double-blind, multicenter, 2 arms	To assess whether supplementation with omega 3 long-chain polyunsaturated fatty acids, administered from early pregnancy (<20 weeks of gestation) until 34 weeks of gestation, would result in a lower incidence of early preterm delivery than control, without increasing the incidence of post term obstetrical interventions, in a broad population of pregnant women with a single fetus or multiple fetuses	6centers in four states in Australia	A total of 5544 pregnancies in 5517 women were randomly assigned to:<br>Group 1: omega 3 long-chain polyunsaturated fatty acid capsules that provided approximately 800 mg of DHA and approximately 100 mg of EPA (2770 pregnancies in 2766 women).<br>Group 2: control group, vegetable-oil capsules that provided a total of approximately 15 mg of DHA and approximately 4 mg of EPA per day (2774 pregnancies in 2765 women)<br>A total of 5431 pregnancies (2704 in group experimental and 2724 in group placebo) completed the study.	From the time of trial entry (<20 weeks of gestation) until 34 weeks of gestation or until delivery, whichever occurred sooner.	Preterm delivery <37 weeks, preterm delivery <34 weeks, preeclampsia, small for gestational age, neonatal death, intrauterine fetal death.	Mid, 800 mg of DHA, 100 mg of EPA

**Malcolm 2003 [45]**	RCT, double-blind, placebo-controlled,2 arms	To test the hypothesis that the dietary supplementation with fish oil rich in DHA enhances retinal function of their infants as measured by the electroretinogram in healthy pregnant women with singleton pregnancies.	Yorkhill NHS Trust, Glasgow, UK	100 women were randomized to receive dietary supplementation with two fish oil capsules per day, that provided 100 mg of DHA per capsule (experimental group, 50 women) or sunflower oil capsules (control group 50 women). A total of 63 pregnancies completed the study until delivery (31 in group experimental and 32 in group placebo).	From enrollment (approximately 15 weeks of pregnancy) until the time of delivery.	Preterm delivery <37 week.	Low, 200 mg of DHA, not reported EPA
**Mardones 2007 [46]**	RCT, non-blinded, multicenter, 2 arms	To test the hypothesis that food fortification with omega 3 fatty acids and multiple micronutrients increases birth weight and gestation duration in healthy pregnant women with singleton pregnancies	19 urban health clinics, Southeast Metropolitan Public Health Service, Santiago, Chile	1173 women were assigned to receive regular powdered milk (control group, 552 women) or a milk product fortified with multiple micronutrients and omega 3 fatty acids (experimental group, 598 women) that provided 0.54 g of DHA and 2.6 g of omega-6 LCPUFA. 698 pregnancies (365 in group experimental and 333 in group placebo) completed the study until delivery. 32 women were excluded post randomization.	From enrollment (<20 weeks of pregnancy) until delivery.	Preterm delivery <37 weeks, preterm delivery <34 weeks, preeclampsia, small for gestational age.	Low, 540 mg of DHA
**Miller 2016 [47]**	RCT, double-blind, placebo-controlled, 2 arms	To examine the effects of omega 3 DHA intake during pregnancy and lactation on infant neuro cognitive development using the BSID-III during the first year of life in healthy pregnant women with singleton pregnancies	Private practice gynecology and obstetrics clinics in the Fort Collins, Colorado	115 women were randomized to receive a supplement of highly purified tuna fish oil containing 300 mg DHA and 67mg EPA prepared as one hard capsule (experimental group, 60 women) or an identical high oleic acid sunflower oil placebo (control group, 55 women).<br>A total of 112 pregnancies (59 in group experimental and 53 in group placebo) completed the study until delivery	From the last trimester of pregnancy through the first 3months of lactation.	Preterm delivery <37 weeks	Low, 300 mg of DHA, 67 mg EPA

**Min 2014 [48]**	RCT, double-blind, placebo-controlled, 4 arms	To investigate if DHA-enriched fish oil supplementation rectifies the red cell membrane anomaly in women with Type 2 diabetes with singleton pregnancies	Antenatal clinic, Newham University Hospital, London, UK.	173 women, 88 with type 2 diabetes and 85 with uncomplicated pregnancy, were randomized to either fish oil or placebo. The experimental group included 86 women (41 with type 2 diabetes and 45 healthy women) who received 2 capsules per day equivalent to 600 mg DHA and the placebo group included 87 women (47 with type 2 diabetes and 40 healthy women) who received 2 capsules per day of sunflower oil equivalent to 82.6% oleic acid. A total of 117 pregnancies (60 in fish oil group and 57 in placebo group) completed the study until delivery	From the first trimester of pregnancy until of delivery.	Preterm delivery <37 weeks, preterm <34 weeks, fetal death.	Mid 600 mg DHA; EPA not reported.
**Min 2016 [49]**	RCT, double-blind, placebo-controlled, 2 arms	To measure the red blood cell membrane phospholipid DHA levels in the women with type 2 diabetes with singleton pregnancies and their neonates at delivery.	Newham University Hospital, London	138 women were randomized to receive daily 2 capsules of either "DHA-enriched formula" (n=67) or "placebo high oleic acid sunflower seed oil" (n=71). Each active supplement capsule contained 300 mg of DHA, 42 mg of EPA and 8.4 mg of AA, and placebo 721 mg of oleic acid.<br>A total of 138 women were randomized in the study. A total of 114 pregnancies (58 in experimental group and 56 in placebo group) completed the study until delivery.	From the recruitment (17 - 33 gestational weeks) until delivery.	Preterm delivery <37 weeks, preterm <34 weeks, fetal death.	Mid: 600 mg DHA, 84 mg EPA

**Mozurkewich 2013 [50]**	RCT, double-blind, placebo-controlled, 3 arms.	To test the effects of EPA and DHA rich fish oils on prevention of depressive symptoms among women with single pregnancy at an increased risk of depression.	University of Michigan Health System and St Joseph's Mercy Hospital Health System, Southeastern Michigan, USA	126 women were randomized to receive EPA-rich fish oil (42 women, 1060 mg EPA plus 274 mg DHA), DHA-rich fish oil (42 women, 900 mg DHA plus 180 mg EPA), or soy oil placebo (42 women). A total of 126 women were randomized in the study. A total of 42 women were assigned to EPA-rich fish oil, 42 women were assigned to DHA-rich fish oil and 42 women were assigned to placebo. 118 women completed the study until delivery (39 in the group EPA-rich fish oil, 38 in the group DHA-rich fish oil and 41 in placebo group)	From the enrollment (12 - 20 gestational weeks) until 6 - 8 weeks postpartum	Preeclampsia	Group EPA-rich fish oil: High: 274 mg DHA + 1060 mg EPA. Group DHA-rich fish oil: High: 900 mg DHA + 180 mg EPA
**Olsen 1992 [51]**	RCT, 3 arms.	To test the effects of a fish oils supplementation on pregnancy duration, birthweight, and birth length in healthy pregnant women with singleton pregnancies.	Midwife clinic in the city of Aarhus, Denmark	533 women were randomized to receive fish oil supplement (n=266), olive oil (n=136), or no supplement (n=131). The fish oil supplement was four 1 g gelatin capsules daily containing fish oil (Pikasol: 32% EPA, 23% DHA, and 2 mg tocopherol/ml); this corresponds to about 2.7 g omega 3 fatty acids per day. 533 women completed the study until delivery	From the enrollment (30th week of gestation) until of delivery.	Preterm <37, intrauterine fetal death.	High: 621 mg DHA + 864 mg EPA

**Olsen 2000 [10]**	RCT, multicenter, consisting of a series of prophylactic and therapeutic trials, 2 arms	To test the preventive effects of dietary omega 3 fatty acids on preterm delivery, intrauterine growth retardation, and pregnancy induced hypertension in women with high-risk pregnancies.	Nineteen hospitals in Europe (Denmark, Scotland, Sweden, England, Italy, The Netherlands, Norway, Belgium and Russia).	1647 women were randomized to receive fish oil (n=818) or olive oil (n=829). The treatment was fish oil (Pikasol: 32% EPA, 23% DHA, and 2 mg tocopherol/mL,) whereas the controls received olive oil (oleic acid [18:1 n-91 72%, linoleic acid [18:2: n-61 12%). In the four prophylactic trials four capsules of either oil were given per day, while in the two therapeutic trials nine capsules were given per day. For women randomized to fish oil, these amounts correspond to 2.7 g (1.3 g EPA and 0-9 g DHA) and 6.1 g (2.9 g EPA and 2.1 g DHA) of long-chain omega 3 fatty acids per day in the prophylactic and therapeutic trials, respectively.	Prophylactic trials: from the 20 weeks until of delivery. Therapeutic trials: from the 33 weeks of gestation until of delivery.	Preterm delivery <37 weeks, preterm <34 weeks, fetal death, preeclampsia, small for gestational age.	Prophylactic trials: high: 900 mg DHA plus 1300 mg EPA. Therapeutic trials: high: 2100 mg DHA plus 2900 EPA.
**Olsen 2019 [15]**	RCT, multicenter, 3 arms.	To investigate whether there is a preventive effect of omega 3 PUFAs on preterm birth in healthy pregnant women with singleton pregnancies.	Lan-Zhou City, Zhang-Ye City, Bai-Yin City, Xi'an City and Wei-Nan City	5531 women were randomized to receive high fish oil supplement (n=1706), low fish oil supplement oil (n=1695), or olive oil (n=1717), providing a total of 2.0, 0.5, and 0 g/d of omega 3 LCPUFAs, respectively. 413 could not be included in the analyses.	From the enrollment (30th week of gestation) until 259 days of gestation.	Preterm < 37, intrauterine fetal death, neonatal death.	High fish oil supplement: mild 440 mg DHA + 660 mg EPA. Low fish oil supplement: low 110 mg DHA + 165 mg EPA
**Onwude 1995 [52]**	RCT, double-blind, placebo-controlled, 2 arms	To investigate whether omega 3 fatty acids (EPA/DHA) would reduce the occurrence of proteinuric and nonproteinuric pregnancy induced hypertension and asymmetrical growth retardation in women with singleton pregnancies at relatively high risk for these disorders	Antenatal clinic of St James's University Hospital, Leeds.	233 women were randomized to receive daily 9 capsules of 2.7 g MaxEpa containing 180 mg EPA per capsule plus 120 mg DHA per capsule (experimental group,113 women) or matching air-filled capsules (control group, 119 women). 232 pregnancies (113 in group experimental and 119 in group placebo) completed the study	From the recruitment (mean of 24 gestational weeks) until the 38th week of pregnancy.	Preterm delivery <37 weeks, preeclampsia, small for gestational age, intrauterine fetal death, neonatal death.	High: 1080 mg DHA, 1620 mg EPA

**Pellonperä 2019 [53]**	RCT, double-blind, placebo-controlled, 4 arms	To assess whether the risk of GDM may be lowered, and glucose metabolism improved by daily administration of fish oil and/or probiotic supplements in overweight and obese pregnant women with singleton pregnancies	Turku University Hospital and University of Turku in Finland	A total of 439 women were randomized in the study. A total of 109 women were assigned to "fish oil + placebo" group, 110 women to the "probiotics + placebo" group, 109 women to the "fish oil + probiotics" and 110 to the "placebo + placebo". The fish oil capsules contained a total of 2.4 g of omega 3 fatty acids, of which 79% (1.9 g) was DHA and 9.4% (0.22 g) EPA. The probiotic capsules contained Lactobacillus rhamnosus HN001 and Bifido-bacterium animalis ssp. lactis 420, each 1010 colony-forming units per capsule. 377 pregnancies (96 in group "fish oil + placebo", 99 in group "probiotics + placebo", 91 in group "fish oil + probiotics" and 91 in group "placebo + placebo") completed the study.	From the first study visit (mean of 13.9 gestational weeks) until 6 months postpartum.	Preterm delivery <37 weeks, preeclampsia, small for gestational age, intrauterine fetal death.	High: 1900 mg DHA, 220mg EPA
**Ramakrishnan 2010 [54]**	RCT, double-blind, placebo-controlled, 2 arms	To evaluate whether prenatal DHA supplementation increases gestational age and birth size in healthy pregnant women with singleton pregnancies.	Mexican Institute of Social Security (IMSS) General Hospital I and three small health clinics within the IMSS system in Cuernavaca Cuernavaca, Mexico	1094 women were randomized to take two capsules daily of algal DHA (547 women) or two capsules daily of placebo (547 women). The DHA capsules contained 200 mg of DHA, and the placebo capsules contained olive oil. 973 women (485 in group DHA and 488 in group placebo) completed the study	From 18 to 22 weeks of gestation through delivery	Preterm delivery <37 weeks, small for gestational age, intrauterine fetal death, neonatal death.	Mid: 400 mg DHA, not reported EPA

**Razavi 2017 [55]**	RCT, double-blind, placebo-controlled, 4 arms	To determine the effects of vitamin D and omega-3 fatty acids supplementation on biomarkers of inflammation, oxidative stress and pregnancy outcomes in GDM patients.	Iran	120 women were randomly assigned into four groups to take either 1000 mg omega-3 fatty acids containing 180 mg EPA and 120 mg DHA twice a day + vitamin D placebo (n = 30) or 50,000 IU vitamin D every 2 weeks + omega-3 fatty acids placebo (n = 30) or 50,000 IU vitamin D every 2 weeks + 1000 mg omega-3 fatty acids twice a day (n = 30) or vitamin D and omega-3 fatty acids placebos (n = 30) for 6 weeks. The placebo capsule contained 500 mg of liquid paraffin. 120 women completed the study.	From 24–28 weeks of gestation for 6 weeks.	Preterm delivery <37 weeks, preeclampsia.	Mid: 240 mg DHA, 360 mg EPA
**Smuts 2003 [26]**	RCT, double-blind, placebo-controlled, 2 arms	To determine the effects of higher intake of DHA an omega 3 LCPUFA on duration of gestation and birth weight in healthy pregnant women with singleton pregnancies.	Prenatal clinic of the University of Missouri-Kansas City and the University of Kansas Medical Center, Kansas City.	350 women were randomly assigned to high-DHA eggs contained a mean of 133 (+/-15) mg of DHA per egg (range 108–165 mg) or ordinary eggs contained a mean of 33 (+/-11) mg of DHA per egg (range 22–51 mg). 176 women were assigned to high-DHA group and 174 to ordinary eggs group. A total of 291 women completed the study	From 24–28 weeks of gestation until delivery.	Preterm delivery <37 weeks, preeclampsia.	Mid: 228 mg DHA, EPA not reported
**Smuts 2003 [56]**	RCT, 3 arms	To test the feasibility of consuming high-DHA hen eggs compared to ordinary eggs to increase blood lipid levels of DHA during the last trimester of healthy pregnant women mainly African American	Regional Medical Center (Memphis, TN), USA	52 women were randomized to two egg groups: 25 to the regular eggs group that contained 18 mg of DHA/egg and 27 to the high DHA egg group contained 135 mg of DHA/egg. Another 21 women consented to the study but were not randomized and were not given eggs (low-egg- intake group). A total of 35 women were analyzed.	During the last trimester of pregnancy, starting between 24 - 28 weeks.	Preterm delivery <37 weeks, preeclampsia.	Low: 135 mg DHA, EPA not reported

**Soldo 2018 [57]**	RCT, single-blind, placebo-controlled, 2 arms	To compare the effect of EPA and DHA dietary supplementation on their concentration in total lipids, and lipid fractions of maternal and umbilical vein blood in healthy pregnant women with singleton pregnancies.	Mostar Clinical Hospital, Department of Gynecology and Obstetrics, Bosnia	90 women were randomized in two groups: supplemented group with EPA and DHA (n=45) and the placebo group (n=45). The supplemented group received 360 mg EPA and 240 mg DHA per day. The placebo group received corn oil. 87 women were included in the analysis	From 14 week of gestation until delivery	Preterm delivery <37 weeks	Mid: 240 mg DHA and 360 mg EPA
**Tofail 2006 [58]**	RCT, double-blind, 2 arms	To test the hypothesis that supplementation with fish-oil (ready form of DHA), during the last trimester of pregnancy, will provide additional DHA to fulfil increased fetal requirement of DHA for developing the nervous system via placental circulation and breastmilk, which will have a positive effect on the mental and psychomotor development of infants in healthy pregnant women.	Dhaka city, Bangladesh	400 women were randomly assigned either to the treatment (fish-oil) or to the placebo group (soy-oil). The treatment group (200 women) received four fish-oil capsules (1 g each) as a single daily dose, equivalent to 1.2 g of DHA and 1.8 g of EPA per day, and the placebo group received an equal number of capsules containing an equal volume of soy-oil (200 women). 324 women were available at delivery	From 25 week of gestation until delivery	Preterm delivery <37 weeks, fetal death and neonatal death.	High: 1.2 g DHA plus 1.8 g EPA
**Van Goor 2009 [59]**	RCT, double-blind, placebo-controlled, 3 arms.	To investigated whether supplementation with DHA during pregnancy and lactation influences the infant's brain development and whether additional AA modulates this effect in healthy pregnant women with singleton pregnancies.	City of Groningen in The Netherlands.	183 women were randomized into 3 groups:<br>1. DHA group (n= 42).<br>2. DHA + AA group (n=41).<br>3. Control group (n=36).<br>Every group received 2 capsules per day, the DHA group received one capsule with 220 mg DHA and one capsule containing soyabean oil, the DHA + AA group received one capsule with 220 mg DHA and other with 220 mg AA and the control group received two capsules containing soyabean oil. 64 women were excluded	From about week 17 of pregnancy until 12 weeks postpartum.	Preterm delivery <37 weeks.	Low: 220 mg DHA, EPA not reported.

post randomization. 119 women were available at delivery

DHA, docosahexaenoic acid; EPA, eicosapentanoic acid; ALA, alpha-linoleic acid; AA, arachidonic acid; LCPUFA, long chain polyunsaturated fatty acid; GLA, Gamma liinolenic acid; GDM, gestational diabetes mellitus.
